# Analysis of Cyanogenic Compounds Derived from Mandelonitrile by Ultrasound-Assisted Extraction and High-Performance Liquid Chromatography in Rosaceae and Sambucus Families

**DOI:** 10.3390/molecules26247563

**Published:** 2021-12-14

**Authors:** Roberto Rodríguez Madrera, Belén Suárez Valles

**Affiliations:** Área de Tecnología de los Alimentos, Servicio Regional de Investigación y Desarrollo Agroalimentario (SERIDA), 33300 Villaviciosa, Asturias, Spain; mbsuarez@serida.org

**Keywords:** cyanogenic compounds, mandelonitrile, amygdalin, prunasin, sambunigrin, HPLC-DAD, UAE, method validation

## Abstract

An analytical method for extraction and quantitative determination of amygdalin, prunasin, and sambunigrin in plant material is described. The method is based on extraction with high-power ultrasound (UAE), with acidified water as solvent and quantification by HPLC–DAD. The best extraction conditions were: 80% sonication amplitude, 55 s extraction time, 70% duty cycle, 0.1 g sample mass, and 10 mL acidified water (0.1% perchloric acid). Once developed, the method was validated in terms of accuracy and precision. Good linearity was obtained, with correlation coefficients exceeding 0.999 and the quantification limits ranged from 2.2 μg/g (amygdalin) to 9.6 μg/g (sambunigrin). The accuracy (recovery study) ranged between 90 and 104% and the reproducibility of the method was always <2.3% (RSD). Special attention should be paid to the ratio sample/solvent in samples with potential β-glucosidase activity to avoid degradation of the cyanogenic glycosides (CNGs). The proposed method was used to evaluate the content of CNGs in kernels of *Prunus* genera, apple seeds, apple pomace, and different plant materials of *Sambucus nigra*.

## 1. Introduction

Cyanogenic glycosides (CNGs) are a group of secondary metabolites of the plant kingdom involved in different functions, such as the transport and turnover of nitrogen and plant development [1].

CNGs are formed by an aglycone, which contains in its structure a nitrile group with a hydroxyl group in the alpha position, to which sugar is attached (generally a monosaccharide or a disaccharide). CNGs release hydrogen cyanide (HCN) in the presence of β-glucosidase and α-hydroxynitrile lyase enzymes [2] in a process known as cyanogenesis [3]. HCN is a highly toxic compound and, therefore, the presence of CNGs is considered as part of the defense system of plants against attack by herbivores [4].

The European Food Safety Authority (EFSA) has carried out an exhaustive study on the identification and characterization of the risks and health hazards of cyanide [5]. This study highlights that cyanide is of high acute toxicity in humans. The lethal dose is reported to be 0.5 to 3.5 mg/kg of body weight. In Europe, the presence of cyanide in food, beverages, and additives is regulated by various standards [6,7,8]. A maximum level of HCN of 50 mg/kg has been established in nougat, marzipan or their substitutes or similar products, 5 mg/kg in canned pitted fruits and 35 mg/kg and a maximum HCN content of 7 g/hL at 100% volume alcohol in fruit marc brandy and stone fruit brandy. Cyanogenic glycosides are mentioned in the legislation of some countries, such as the United Kingdom [9], Germany [10], Australia, and New Zealand [11].

Due to their potential toxicity, several studies have focused on the presence of CNGs in different plants, and they have been reported in over 2500 plant species [3,12]. CNGs are found in different parts of plants (seeds, leaves, stems, fruits) and are derived mainly from the amino acids tyrosine, valine, leucine, isoleucine, and phenylalanine [13].

In this regard, the mandelonitrile derivatives, naturally synthesized from phenylalanine, are one of the most widespread groups of CNGs. Thus, amygdalin or (R)-mandelonitrile 6-*O*-β-D-gentiobioside and R-prunasin or (R)-mandelonitrile β-D-glucoside have been detected in seeds and kernels of members of the Rosaceae family, such as apple, almond, cherry, apricot, and many others [14,15,16]. On the other hand, sambunigrin or S-prunasin is a major cyanogenic compound in the leaf of *Sambucus nigra*, and it is present in other edible parts of the species [17].

It is possible to determine the total content of CNGs in plant samples by indirect methods, such as the picrate method [18], in which, after hydrolysis, the total HCN content is estimated. Cho et al. [19], however, estimated the total HCN content by ion chromatography. 

The analytical methods described in the literature to quantify CNG from mandelonitrile usually employ water, methanol or their mixtures, and different extraction systems such as Soxhlet extraction, solid-phase extraction, ultrasound bath or solid–liquid extraction assisted by microwaves [1,2,14,17,20,21,22] during extraction periods that may last for 24 h. The diverse chemical nature of cyanogenic glycosides means that the extraction and analysis of individual compounds can be difficult and furthermore, degradation can be rapid under appropriate conditions [16].

Ultrasound-assisted extraction (UAE) is a green alternative increasingly employed for the recovery of biomolecules in plant material. The ultrasonic waves generate pressure and cavitation, causing the rupture of the plant cells, which facilitates the extraction of the molecules of interest. Ultrasonic probes are designed to be immersed in the sample, and deliver the ultrasonic intensity directly to the liquid sample, which enhances the extraction yields and reproducibility and reduces the extraction time [23]. Compared with other methods, UAE is a fast technique, which is of particular interest in order to avoid the hydrolysis of the CNGs.

Extracts containing CNG are usually analyzed by reverse phase HPLC with UV–Vis detectors [16], HPLC-MS [21], or tandem HPLC-DAD-MS [17]. The separation of the R and S epimers has been found to be one of the main difficulties of these methods, for which reason some authors have proposed the use of chiral phases [24].

The objective of this work has been the development and validation of an analytical method for the quantitative determination of the main cyanogenic derivatives of mandelonitrile (amygdalin, prunasin, and sambunigrin) by means of HPLC/DAD. UAE conditions have been optimized by using response surface methodology. Analytical parameters, such as linearity, limits of detection, quantification, precision, and accuracy were estimated. The proposed method has been used to evaluate the content of CNGs in kernels of *Prunus* genera, apple seeds, apple pomace, and different plant materials of *Sambucus nigra*.

## 2. Results and Discussion

### 2.1. Chromatographic Separation and Identification

The optimization of chromatographic conditions was carried out with extracts obtained according to the preselection of extraction conditions subsection.

**Figure 1:**
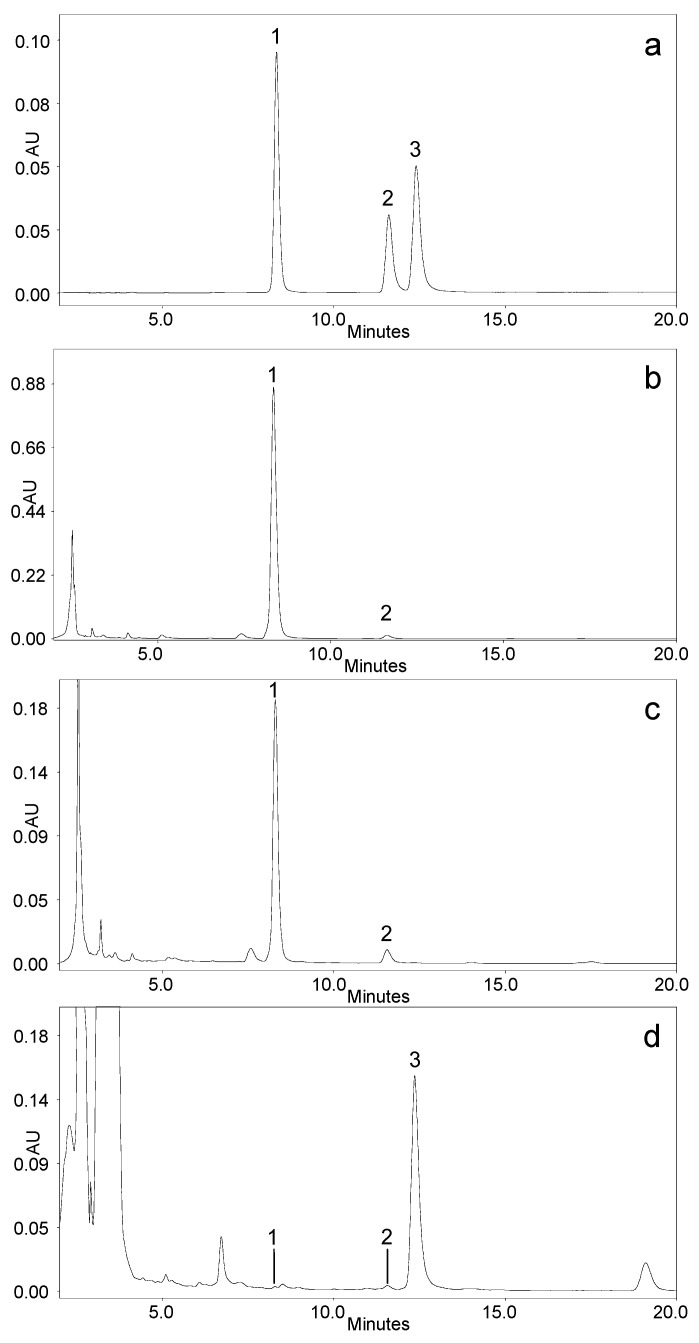
Chromatographic separation of cyanogenic glucosides: (**a**) mixture containing pure standards of amygdalin (1), prunasin (2) and sambunigrin (3), (**b**) plum kernel, (**c**) apple seed, (**d**) Sambucus nigra leaf.

One of the main difficulties in the analysis of cyanogenic derivatives of mandelonitrile is the separation of the diastereoisomers prunasin and sambunigrin from one another. To this end, the conditions employed by Bolarinwa et al. [16] were used as the point of departure, and a column temperature range of between 25 and 40 °C was studied. The best separation conditions were obtained at 25 °C, in an isocratic mode with a mobile phase consisting of 25% methanol in water at a flow of 1.0 mL/min. Figure 1 displays typical chromatograms of a mixture of standards and different extracts containing the analytes of interest. As seen in Figure 1, the three peaks are well separated, with a resolution> 2.0 in the case of the prunasin isomers.

The identification of the peaks was carried out by comparing the retention times and their UV–Vis spectra with those of the pure standards. In all the samples analyzed, the spectra of the compounds of interest did not present contamination and can be considered pure according to the purity criteria implemented in the Empower 3.0 program (purity angle < purity threshold).

### 2.2. Extraction Optimization

#### 2.2.1. Preselection of Extraction Conditions

According to the concentrations described in the literature, a 1/100 ratio (0.1 g sample and 10 mL of solvent) was established as appropriate to perform the extraction quantitatively and efficiently in a single extraction. In a similar way, extraction conditions using UAE for 2 min with interval pulses of 20% and maximum amplitude (100%) were chosen for the initial experimentation.

Under these conditions, extractions were carried out with different methanol/water mixtures (100%, 70%, 50%, 30%, 10%, and 0%) and water with 0.1% perchloric acid on two matrices: apple seed and leaves of *S. nigra*.

**Figure 2:**
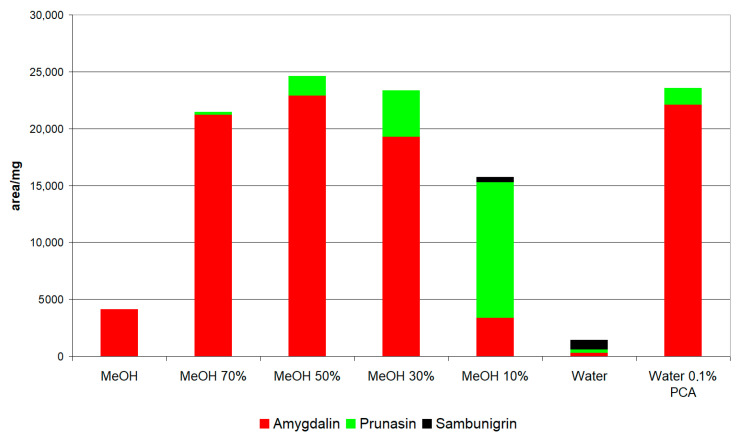
Extraction efficiency with different solvents in apple seed. PCA: perchloric acid.

Figure 2 shows the extraction from apple seed with different solvents. As can be seen, the extraction of amygdalin in methanol mixtures was greater in a 50% mixture and at a higher proportion of water the recovery of amygdalin decreases and the content of prunasin and sambunigrin increases. It is important to highlight that sambunigrin has not been described in apple seed, but nonetheless, our results show that this compound can be formed and that the highest concentration of sambunigrin is detected when the solvent is water. In this sense, several authors have indicated that the degradation of amygdalin is closely linked to the extraction conditions [24,25].

When extraction is carried out with acidified water (0.1% perchloric), the content of amygdalin and prunasin is similar to the maximum detected for the methanol/water mixtures (Figure 2). Therefore, it was decided to carry out the extractions in water with 0.1% perchloric acid, which avoids the use of organic solvents.

No significant differences were detected in the *S. nigra* leaf extracts with the different solvents, the sambunigrin extraction being similar in all cases.

#### 2.2.2. Optimization of the Extraction Condition by UAE

The extraction was optimized on the two matrices indicated above: apple seed and *S. nigra* leaf with 0.1 g of mass and 10 mL of water with 0.1% PCA.

The UAE conditions were optimized following a Box–Behnken design with three factors. Table 1 shows the extraction conditions and the area/mg values obtained for amygdalin in apple seed and sambunigrin in *S. nigra* leaf according to the BBD.

Table 2 shows the results of fitting the second-order polynomial models for these variables. ANOVA indicated that both the models were statistically significant by the probability of the F-test at a level below 0.01. Moreover, the probability values of the lack of fit tests were not significant in all cases (*p* > 0.10), indicating that the regression models are a good prediction of the experimental results. Satisfactory determination coefficients were obtained for both analytes (Table 2). Adjusted R^2^, 0.859 for amygdalin and 0.895 for sambunigrin, imply that the major percentage of variance of these variables is explained by the respective models, while the difference between these values and the predicted R^2^ show that the models are not overfitted. The degree of precision, estimated as relative standard deviation (RSD) was very reliable, with values lower than 2%.

**Figure 3:**
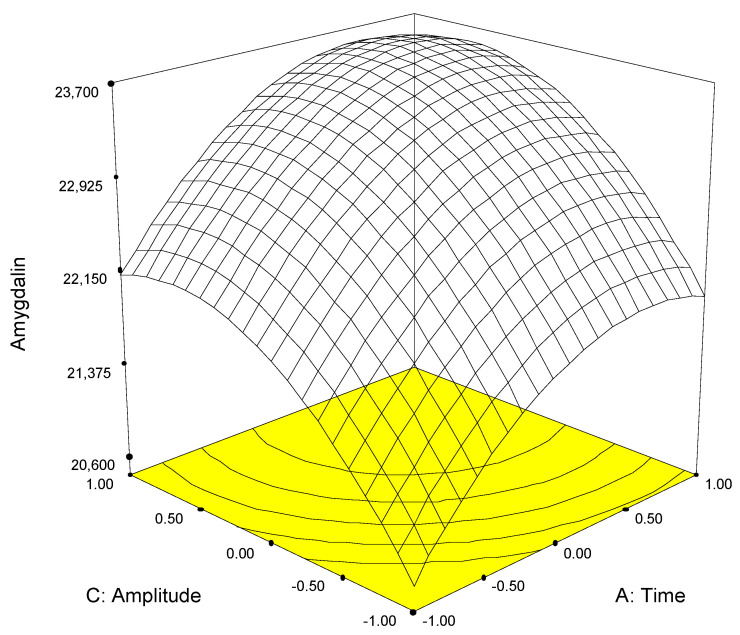
Response surface calculated from experimental data for amygdalin in apple seed slurry.

**Figure 4:**
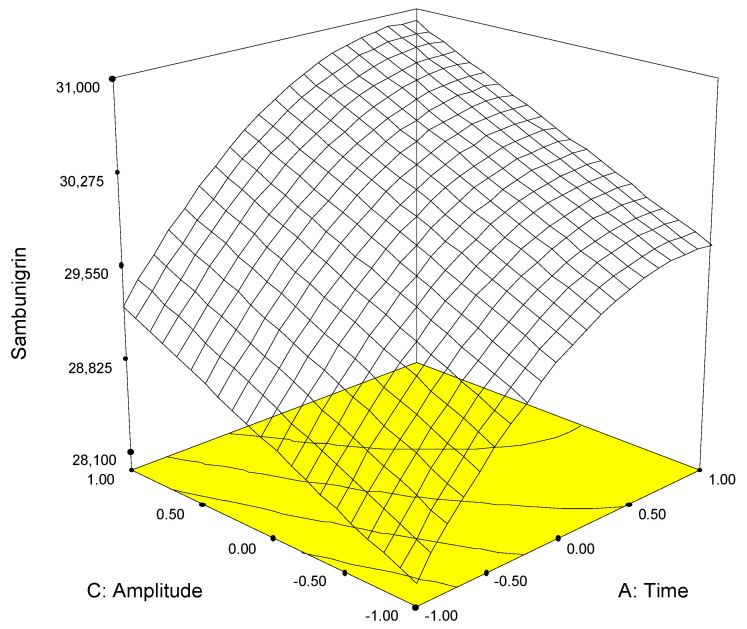
Response surface calculated from experimental data for sambunigrin in *S. nigra* leaf.

The physicochemical properties of amygdalin and sambunigrin are similar, since they are both saccharides (gentiobiose or glucose) linked to the same aglycone (mandelonitrile). Furthermore, they are soluble in water, so it seems reasonable that the models built for each of the analytes should be similar (Figure 3 and Figure 4). Thus, for both analytes, similar models were obtained in which the significant first-order factors were time and amplitude but not the frequency with which the pulses are emitted (Table 2), while the differences are found in the second order terms (quadratic terms and interactions).

Since the mathematical models were similar for both variables, it was possible to establish—as a requirement for obtaining the optimal extraction results—that these were the conditions that simultaneously maximized the recovery of each analyte. Among the range of conditions satisfying this requisite, those selected were as follow: time 55 s, pulse 70%, and amplitude 80%.

Although the extraction conditions were optimized for a 1/100 sample/solvent ratio, the possibility of using different ratios in the matrices of interest was evaluated.

**Figure 5:**
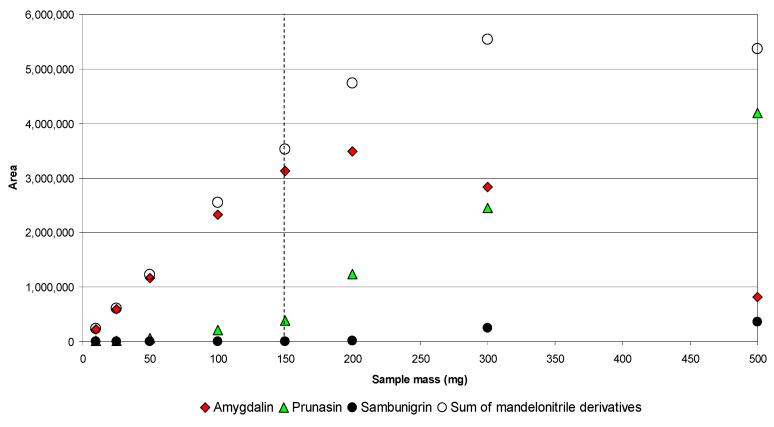
Extraction of amygdalin, prunasin, sambunigrin, and sum of mandelonitrile derivatives in apple seed at different sample mass.

For this, the sample mass was varied between 0.01 and 0.5 g, keeping a fixed volume of 10 mL as it was a recommended working volume for the sonotrode used (diameter 2 mm). The behavior was different in the evaluated matrices. Thus, while in *S. nigra* leaves the response for sambunigrin was proportional to the mass in the entire range evaluated (R = 0.9995, Supplementary Figure S1), in the case of apple seed, the efficiency of the extraction of amygdalin was linear only in the range 0.01–0.15 g (R = 0.994, Figure 5). In addition, the lower extraction of amygdalin was accompanied by an increase in the percentage of prunasin and sambunigrin in the extracts, which indicates the degradation of amygdalin by hydrolysis of the β (1–6) bond of gentiobiose and the formation of the two monoglycosidic epimers. Likewise, as can be seen in Figure 5, the sum of the three mandelonitrile CNGs ceases to be linear from a mass of 0.2 g (ratio 1/50, r = 0.998), which shows that, in addition to hydrolysis of the β (1–6) bond of gentiobiose, subsequent hydrolysis of the glycosidic bonds of prunasin and sambunigrin takes place. Based on these results, it can be established that, in the case of apple seed, ratios greater than 1/66 may lead to underestimation of the content of amygdalin, as a result of its hydrolysis and the formation of the epimers prunasin and sambunigrin, while ratios greater than 1/50 give rise to underestimated values for total mandelonitrile CNGs.

### 2.3. Validation Procedure

The model was validated experimentally by two operators, on different days and conducting the tests independently (three replicates per operator/day).

Predicted and experimental values for variables are displayed in Table 3. The results were found within the predicted intervals, and the average accuracy of the values obtained against those predicted was 97% for amygdalin and 102% for sambunigrin.

#### 2.3.1. Precision, Accuracy, and Extract Stability

Precision was calculated in two ways: repeatability and reproducibility. Repeatability (r), estimated for each analyst and compound ranged between 0.4–4.1% and reproducibility (R), evaluated as the RSD between analysts was in all cases ≤2.3% (Table 3).

The accuracy of the method was evaluated in two ways: by exhaustive extraction of the plant material and in a recovery study conducted by spiking both matrixes with a known concentration of standard. 

**Figure 6:**
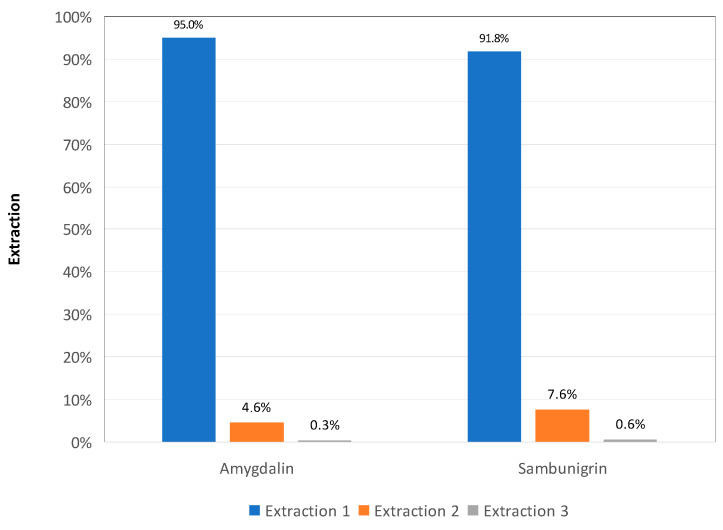
Result of the sequential extraction of amygdalin in apple seed and sambunigrin in *S. nigra* leaf and the percentage extracted in each one of them.

Exhaustive extraction was carried out through four consecutive extractions from each sample involved in the validation (apple seed and *S. nigra* leaves). In this case, accuracy was estimated as area percentage (%) in the first extraction. The values obtained showed a total accuracy of 95% for amygdalin in apple seed, and 92% for sambunigrin in *S. nigra* leaves (Figure 6).

Recovery values between 100% and 104% for amygdalin in apple seed and between 90% and 92% for sambunigrin in *S. nigra* leaves were obtained. Furthermore, the comparison of the slopes of the calibration lines and the addition lines, by means of a *t*-student test for non-homogeneous variances, did not show significant differences. Supplementary Figure S2 shows the slopes of the calibration and addition lines for amygdalin and sambunigrin.

To evaluate the extracts’ stability, they were maintained at 10 °C and analyzed on 10 consecutive days. Both extracts were stable, with differences of less than 2% in the analytes of interest.

#### 2.3.2. Calibration Curves, LD, and LQ

Calibration parameters are shown in Table 4. All of the compounds showed good linearity, with regression coefficients > 0.9997 over three orders of magnitude.

In addition, method limits of detection and quantification (expressed as μg/g) were calculated from the instrumental limits of detection and quantification previously estimated [26] and taking the volume of extracts (10 mL) and the mass of samples used in extraction (0.1 g).

The method limit of detection ranged from 0.7 μg/g (amygdalin) to 2.9 μg/g (sambunigrin) and the method limit of quantification ranged from 2.2 to 9.6 μg/g for the same compounds.

Taking into account that the minimal lethal oral dose of hydrogen cyanide is estimated to be 0.5 to 3.5 mg/kg body weight [3], and the data reported by other authors [15,16,17], it can be concluded that the proposed method is sufficiently sensitive for the quantitative determination of these potential toxic phytochemicals.

### 2.4. Cyanogenic Glycosides Contents in the Analyzed Samples

#### 2.4.1. Rosacea

The optimized and validated method was applied to analyze a set of different plant materials containing cyanogenic derivatives of mandelonitrile (Table 5). Rosaceae are one of the most interesting plant families in terms of the presence of mandelonitrile CNGs, especially amygdalin and prunasin, in their seeds and kernels. Therefore, the ability of the method to quantify these compounds in the seeds of apples, apple pomaces, and in the kernels of different species of the genus *Prunus* was evaluated.

The highest content of amygdalin was detected in plum kernels (cv. Reina Claudia Verde), with 57.3 mg/g dry sample, in contrast to the 3.6 mg/g detected in prune kernel. High levels of amygdalin were also detected in varieties of *P. persicum* (peach, flat peach, and nectarine) and in *P. armeniaca* (apricot), while the *P. avium* (cherry) samples showed the lowest amygdalin content in this genus, with values between 8.2 and 12.6 mg/g (Table 5).

The values reported for kernels of the analyzed species present wide variability, which can be attributed to different causes, such as the environmental conditions, conservation of the fruit or genetics. Thus, Bolarinwa et al. [16] reported values for plum kernels of between 0.44 and 17.49 mg/g for distinct varieties, pointing out that amygdalin is the major mandelonitrile CNG in green plums. In the present study, the values were found to be even higher. Several authors have described important differences between bitter and sweet varieties of apricot [22,27,28] with values between undetectable and 70 mg/g, the content of amygdalin being higher in bitter varieties than in sweet ones. High ranges of variability have also been found for amygdalin in cherry, with values between 0.732 [15] and 65 mg/g [29], which places the content of the samples analyzed in this work (8.2–12.6 mg/g) in the middle range. Lee et al. [30] detected an amygdalin content of 23.1 mg/g in peaches, similar to our results for the different varieties of *P. persicum*, which are significantly higher than those reported by Bolarinwa et al. [16] for nectarines and peaches, 0.15 mg and 6.81 mg/g respectively.

In the present study, values for amygdalin that were slightly higher than those detected in cherries were detected in the apple seed samples analyzed, ranging between 13.4 and 18.6 mg/g. Likewise, a wide distribution was reported for the content of amygdalin in apple seeds, with values between 0.24 and 0.70 mg/g measured by Amaya-Salcedo [14], 0.95–3.91 mg/g by Bolarinawa et al. [31], 1.19–1.63 mg/g of fresh weight by Senica et al. [32], and 13 mg/g by Opyd et al. [33]. Senica et al. [34] evaluated the cyanogenic glycosides in Golden Delicious seeds from four countries in Eastern Europe, observing that their contents vary greatly due to environmental conditions and with the time of conservation of the apples in a controlled atmosphere; the amygdalin contents of apple seeds ranged from 0.52 to 0.85 mg/g fresh weight at harvest time.

On the other hand, both the apple seeds and the samples of the genus Prunus presented lower amounts of prunasin than amygdalin, in many cases less by an order of magnitude, with values between 0.3 mg/g in several apple seeds and 2.2 mg/g in a cherry sample (Table 5). These results are in agreement with those reported in the bibliography, although the number of references is small [15,22]. However, Senica et al. [34] showed that the main cyanogenic glycoside in Golden Delicious seeds from Azerbaijan and Russia was prunasin (1.32 mg/g and 0.91 mg/g).

Different studies have been carried out on the composition and properties of apple pomace, revealing that it is an interesting raw material due its content in nutrients, phytochemicals, and functional components [35,36]. Apple pomace produced by the cider making industry contains apple seeds in variable quantities, which can account for up to 5% of the total weight [37]. Regarding the apple pomace samples analyzed (Table 5), the content of amygdalin (0.1 mg/g) was especially low and could generate 5.9 µg cyanide equivalents per gram of apple pomace. Acute cyanide toxicity can occur in humans at doses between 0.5 and 3.5 mg/kg body weight [3], so the results for the amygdalin content of apple pomaces guarantee the safe use of this by-product in food formulations.

#### 2.4.2. Sambucus nigra

The species *S. nigra* has been described as potentially toxic due to the content of sambunigrin in its different parts. In this case, the quantification method identified the highest content in the leaves of the species, between 16.2 and 20.7 mg/g dry matter, while the flowers, stalks, and fruits presented significantly lower values of sambunigrin (Table 5). Values for sambunigrin reported by other authors range between 1.0 mg/g dry matter and 0.03–0.21 mg/g fresh sample [17,38]. On the contrary, in this species, the contents of amygdalin and prunasin were unimportant in all parts except the leaves.

## 3. Materials and Methods

### 3.1. Sample Material and Preparation of the Extracts

The fruits (cherry, apricot, peach, flat peach, nectarine, plum) came from the local market. The stones were removed and broken to obtain the intact seeds.

Five apple pomace samples, obtained from the local cider industry, were freeze-dried. The seeds of the apple pomaces were separated manually.

Leaves, flowers, branches, and fruits of *S. nigra* were collected from wild specimens of this species located at the SERIDA facilities.

All samples were freeze-dried and stored under vacuum until the moment of their analysis.

Freeze-dried samples were milled at the time of extraction in a mortar and the powders were sieved through a standard sieve (number 18, corresponding to a sieve open ring size of 1.00 mm).

### 3.2. Reagents and Standards

Amygdalin (CAS number: 29883-15-6) was purchased from Sigma-Aldrich (St. Louis, MO). Prunasin (CAS number: 99-18-3) and sambunigrin (CAS number: 99-19-4) were supplied by LGC standards (Teddington, Middlesex, UK). Methanol HPLC grade was purchased from JT Baker (Deventer, Holland). Water was purified using a Milli-Q system from Millipore (Bedford, MA, USA).

### 3.3. Chromatographic Separation and Identification

Analyses were performed with a Waters system, equipped with a 717 plus autosampler, provided with a temperature controller, a model 600 pump, a diode array detector (DAD 2996) and Empower software v.3.0 data module. Separation of polyphenols was carried out on a reversed-phase Macherey-Nagel Nucleosil 120 C18 column (250 × 4.6 mm I.D, 3 μm) from Fisher Scientific (Loughborough, UK). The column was thermostated at 25 °C and the injection volume was 20 μL. The elution solvent was methanol/water (25/75) in isocratic mode at 1 mL/min. The UV–Vis spectra were acquired from 190 nm to 400 nm with a sampling rate of 1.0 and the highest scanning resolution (1 nm).

The identity of CNGs was ascertained using data from DAD, by comparison and combination of their retention times and UV–Vis spectra and confirmed with authentic standards. Quantification was performed by HPLC–DAD according to an external standard method at 214 nm.

### 3.4. Experimental Design and Extraction Optimization

Ultrasonic assisted extraction (UAE) was employed to extract CNGs. The samples were extracted in a water bath at 20 °C. The extraction was carried out with an UP200Ht ultrasonic homogenizer (Hielscher, Teltow, Germany) equipped with a 2 mm diameter sonotrode at a frequency of 26 kHz and 200 W power input. The extraction was carried out in pulse mode.

A Box–Behnken design (BBD) was applied to maximize the extraction of CNGs. Three factors in the design were studied at three levels (−1, 0, and +1) and five center points to allow efficient estimation of the first- and second-order coefficients. The factors considered to determine the conditions for maximizing extraction of CNG compounds were amplitude of sonication (20, 60, and 100%), time of extraction (20, 40, and 60 s), and duty cycle (20, 60 and 100%).

According to this experimental design (BBD), 17 extracts were carried out. The experimental data were examined using response surface methodology (RSM). The three variables mentioned above were tested for possible linear and quadratic models to find the model with the best fit. To compute the predicted responses (Y) a polynomial Equation (1):

Y = b_0_ + b_1_X_1_ + b_2_X_2_ + b_3_X_3_ + b_12_ X_1_X_2_+ b_13_ X_1_X_3_+ b_23_ X_2_X_3_+ b_123_ X_1_X_2_X_3_+ b_11_X_1_^2^ + b_22_X_2_^2^ + b_33_X_3_^2^ (1)

for each variable was estimated: X_1_ = extraction time, X_2_ = amplitude, X_3_ = duty-cycle, b_0_ is the offset term, b_1_, b_2_ and b_3_ the first order coefficients, b_11_, b_22_, and b_33_ the second order coefficients, and b_12_, b_13_, b_23_, and b_123_ the interaction coefficients.

Design Expert 7.0.0 Software (Stat-Ease, Inc., Minneapolis, MN, USA) was used to analyze the results.

### 3.5. UAE Conditions

The optimum conditions were as follow: amplitude 80% (16 W power output), extraction time 55 s, duty cycle 70%, sample mass 0.1 g and extract volume 10 mL.

After sonication, the samples were immediately filtered through a 0.45 µm cellulose acetate syringe prior HPLC analysis. Two extractions were carried out for each sample.

### 3.6. Validation Procedure

Parameters checked for method validation were selectivity, examining chromatographic blanks and purity criteria of analytes peaks by Empower 3.0; precision, was calculated (RSD %) as repeatability and reproducibility. Accuracy of the method was evaluated by exhaustive extraction (four consecutive extractions of the plant material) and a recovery study conducted by spiking with a known concentration of standard at three levels (low, medium, and high).

The linearity of the response of detector was determined by the square correlation coefficients of the calibration curves generated by injection of standard solutions at concentrations including the range expected in real samples. Instrumental limit of detection and instrumental limit of quantification were estimated as 3 × Sa/m and 10 × Sa/m, respectively, from the residuals of calibration curves at low concentrations, where Sa is the standard deviation of calibration curve intercept values and m is the slope of the calibration curve y = a + mx.

## 4. Conclusions

A method for accurately quantifying the cyanogenic derivatives of mandelonitrile, based on extraction with high-power ultrasound (UAE), with acidified water as solvent and quantification by HPLC–DAD was developed. In this study, the extraction time was significantly reduced when compared to other methods proposed, which is especially important to avoid degradation of amygdalin during the extraction phase. Special attention should be paid to the sample/solvent ratio in samples with potential β-glucosidase activity, such as apple seeds, to avoid the degradation of the cyanogenic glycosides (CNGs) by enzymatic action and, furthermore, ratios greater than 1/50 give rise to underestimated values for all of the mandelonitrile CNGs. The method was successfully applied to characterize cyanogenic derivatives of mandelonitrile in 18 samples of the Rosaceae family and 8 plant materials of *Sambucus nigra*.

## Figures and Tables

**Table 1:** Coded and real values for the experimental factors and area/mg of amygdalin and sambunigrin according to the BBD.

Run	Time (s)	Pulse (%)	Amplitude (%)	Amygdalin (Area/mg)	Sambunigrin (Area/mg)
1	−1 (20)	0 (60)	−1 (20)	21,640	27,098
2	−1 (20)	0 (60)	1 (100)	22,075	29,157
3	1 (60)	0 (60)	−1 (20)	21,495	27,692
4	1 (60)	0 (60)	1 (100)	23,625	29,619
5	0 (40)	−1 (20)	−1 (20)	20,551	28,134
6	0 (40)	−1 (20)	1 (100)	22,408	29,720
7	0 (40)	1 (100)	−1 (20)	22,193	29,577
8	0 (40)	1 (100)	1 (100)	23,123,	30,537
9	−1 (10)	−1 (20)	0 (60)	21,025	29,194
10	1 (60)	−1 (20)	0 (60)	21,457	27,229
11	−1 (20)	1 (100)	0 (60)	22,909	28,600
12	1 (60)	1 (100)	0 (60)	23,246	30,188
13	0 (40)	0 (60)	0 (60)	23,138	29,981
14	0 (40)	0 (60)	0 (60)	23,770	29,791
15	0 (40)	0 (60)	0 (60)	23,549	29,505
16	0 (40)	0 (60)	0 (60)	23,040	30,088
17	0 (40)	0 (60)	0 (60)	22,723	30,575

**Table 2:** Statistics for models constructed from amygdalin and sambunigrin in terms of coded values.

	Amygdalin		Sambunigrin	
Model		***		***
Intercept	23,244.29		29,970.60	
A-Time	668.91	***	816.43	***
B-Pulse	271.72	n.s.	84.87	n.s.
C-Amplitude	753.95	***	578.12	***
AB	423.64	*	-	-
BC	-	-	888.15	***
A^2^	−562.82	*	−455.76	*
B^2^	−472.28	*	−1145.16	***
C^2^	−612.19	**		
Lack of Fit		n.s.		n.s.
R-Squared		0.921		0.935
Adjusted R^2^		0.859		0.895
Predicted R^2^		0.773		0.805
RSD		1.61		1.21

Significance level; *: *p* < 0.05; **: *p* < 0.01; ***: *p* < 0.001; n.s.: not significant.

**Table 3:** Validation of model (time: 55 s; pulse: 70%; amplitude: 80%) under reproducibility conditions.

	Amygdalin (Area/mg)	Sambunigrin (Area/mg)
Prediction	23,770	30,664
95% CI low	23,376	30,299
95% CI high	24,165	31,028
95% PI low	22,860	29,494
95% PI high	24,681	31,533
Operator 1 (*n* = 3)	23,193	31,185
Repeatability, r (RSD)	1.0	4.1
Operator 2 (*n* = 3)	23,062	30,205
Repeatability, r (RSD)	2.0	1.9
Reproducibility, R (RSD)	0.4	2.3

CI: confidence interval. PI: prediction interval; RSD: relative standard deviation.

**Table 4:** Analytical characteristics of the calibration graphs of cyanogenic compounds.

Compound	Calibration Curve				Instrumental		Method	
	Linear Range (mg/L)	Slope	Intercept	Correlation Coefficient	LOD (10^−3^ mg/L)	LOQ (10^−3^ mg/L)	LOD (10^−3^ mg/g)	LOQ (10^−3^ mg/g)
Amygdalin (*n* = 11)	0.24–480	16224	−8999	0.9999	6.5	21.7	0.7	2.2
Prunasin (*n* = 11)	0.19–296	20007	−7906	0.9999	16.1	55.7	1.6	5.6
Sambunigrin (*n* = 10)	0.20–390	22257	−2506	0.9998	28.8	96.0	2.9	9.6

*n* = number of points in calibration curve. LOD: limit of detection. LOQ: limit of quantitation.

**Table 5:** Cyanogenic mandelonitrile derivatives in distinct plant materials (mg/g dry matter).

	Amygdalin	Prunasin	Sambunigrin
*Rosaceae* Kernels			
Cherry-1 (*P. avium*)	12.6	1.8	n.d.
Cherry-2 (*P. avium*)	11.8	2.2	n.d.
Cherry cv picota (*P. avium*)	8.2	1.1	n.d.
Apricot (*P. armeniaca*)	45.0	1.5	n.d.
Peach (*P. persica*)	43.3	0.6	n.d.
Flat peach (*P. persica* var. platycarpa)	23.7	2.0	n.d.
Nectarine (*P. persica* var. nucipersica)	32.7	1.3	n.d.
Plum cv Reina Claudia verde (*P. domestica*)	57.3	0.9	n.d.
Prune kernel (*P. domestica*)	3.6	0.5	0.1
Apple seed			
Golden delicious	13.6	0.9	0.1
Durona de Tresali	14.9	0.3	n.d.
Seed from apple pomace-1	13.4	0.3	n.d.
Seed from apple pomace-2	13.8	0.3	n.d.
Seed from apple pomace-3	15.0	0.5	n.d.
Seed from apple pomace-4	18.6	0.8	n.d.
Seed from apple pomace-5	16.4	1.1	n.d.
Apple pomace flour			
Apple pomace-1	0.1	n.d.	n.d.
Apple pomace-2	0.1	n.d.	n.d.
*Sambucus nigra*			
Young leaf	0.2	0.4	16.2
Old leaf	0.1	0.8	20.7
Flower-1	n.d.	n.d.	0.6
Flower-2	n.d.	n.d.	0.4
Flower bud	n.d.	n.d.	0.3
Stalk	n.d.	n.d.	1.7
Fruit-1 (elderberry)	n.d.	n.d.	0.4
Fruit-2 (elderberry)	n.d.	n.d.	0.3

## Data Availability

Not applicable.

## Supplementary Materials

The following are available online. Figure S1: Extraction of sambunigrin in *S. nigra* leaf at different sample mass. Figure S2: Calibration (▲) and addition (■) lines for amygdalin and sambunigrin.

## Sample Availability

Samples of the compounds are not available from the authors.
