# Supplementary material for: Analysis of Cyanogenic Compounds Derived from Mandelonitrile by Ultrasound-Assisted Extraction and High-Performance Liquid Chromatography in Rosaceae and Sambucus Families
Source: Molecules. 2021 Dec 14;26(24):7563. doi: 10.3390/molecules26247563 (PMC8705553; doi:10.3390/molecules26247563)
Supplement: Supplementary file 1 [file molecules-26-07563-s001.zip › molecules-1482337-supplementary.pdf]

Article

Analysis of Cyanogenic Compounds Derived from Mandelonitrile by Ultrasound Assisted Extraction and High-Performance Liquid Chromatography in Rosaceae and Sambucus Families

Roberto Rodríguez Madrera *, Belén Suárez Valles

Área de Tecnología de los Alimentos, Servicio Regional de Investigación y Desarrollo Agroalimentario (SERIDA), 33300 Villaviciosa, Asturias, Spain; mbsuarez@serida.org
* Correspondence: rrodriguez@serida.org

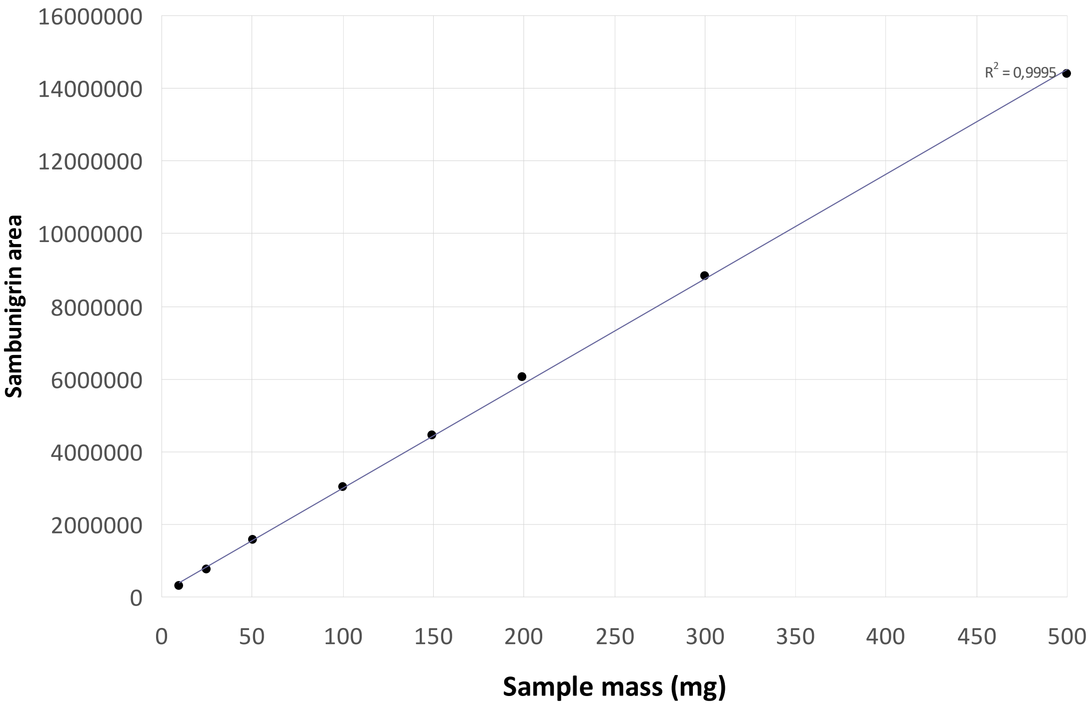

Figure S1. Extraction of sambunigrin in *S. nigra* leaf at different sample mass.

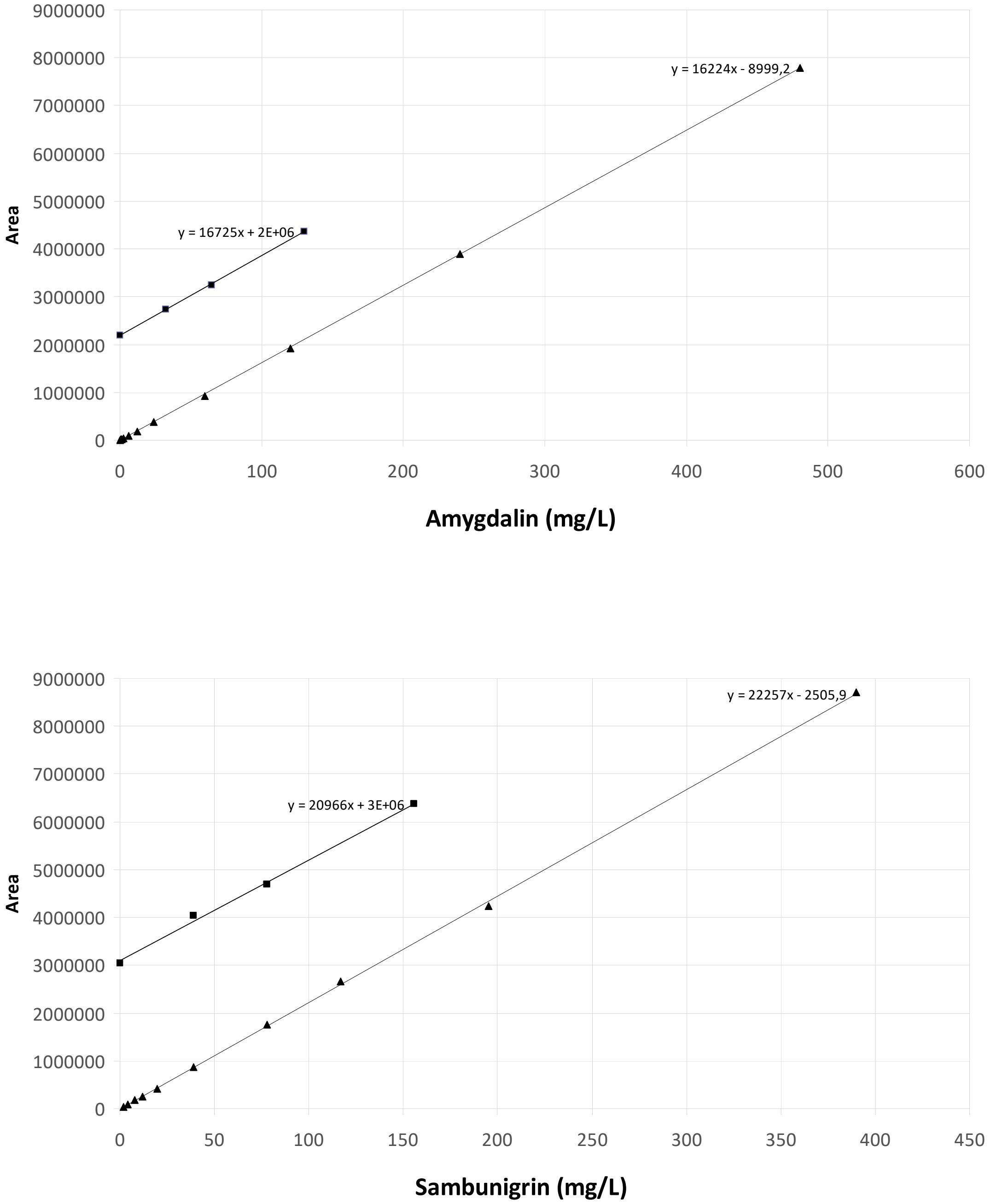

Figure S2. Calibration (▲) and addition (■) lines for amygdalin and sambunigrin.
